# Numerical Study of Paramagnetic Elliptical Microparticles in Curved Channels and Uniform Magnetic Fields

**DOI:** 10.3390/mi11010037

**Published:** 2019-12-28

**Authors:** Christopher Sobecki, Jie Zhang, Cheng Wang

**Affiliations:** Department of Mechanical and Aerospace Engineering, Missouri University of Science and Technology, 400 W. 13th St., Rolla, MO 65409, USA; cas3n3@mst.edu (C.S.); jzn39@mst.edu (J.Z.)

**Keywords:** microparticles, paramagnetic, magnetic field, direct numerical simulation, curved channel, low Reynolds number

## Abstract

We numerically investigated the dynamics of a paramagnetic elliptical particle immersed in a low Reynolds number Poiseuille flow in a curved channel and under a uniform magnetic field by direct numerical simulation. A finite element method, based on an arbitrary Lagrangian-Eulerian approach, analyzed how the channel geometry, the strength and direction of the magnetic field, and the particle shape affected the rotation and radial migration of the particle. The net radial migration of the particle was analyzed after executing a π rotation and at the exit of the curved channel with and without a magnetic field. In the absence of a magnetic field, the rotation is symmetric, but the particle-wall distance remains the same. When a magnetic field is applied, the rotation of symmetry is broken, and the particle-wall distance increases as the magnetic field strength increases. The causation of the radial migration is due to the magnetic angular velocity caused by the magnetic torque that constantly changes directions during particle transportation. This research provides a method of magnetically manipulating non-spherical particles on lab-on-a-chip devices for industrial and biological applications.

## 1. Introduction

Applying magnetic fields to separate magnetic micro- and nanoparticles by shape immersed in a fluid is a long searched for achievement in biomedical and industrial applications, such as cell separation [1,2], drug deliverance [3,4], mining ores [5], and waste management [6]. The method of magnetic separation in these industries stems from the use of magnetophoresis, i.e., magnetic forces. The production of magnetic forces are due to the shape, size, and magnetic susceptibility of the particle and the spatial non-uniform magnetic field [7].

In previous studies, passive methods to separate particles by shape and size have been studied through inertial effects in curved channels by the primary flow (influenced by the Reynolds number) and secondary flow (influenced by the Deans number). Some of the various curved channel designs include single curved [8,9], serpentine [10,11,12], spiral [13,14,15,16,17,18], and wave channels [19,20]. The separation of particles by shape and size are caused by vortices due to the cross-sectional shape of the channel including trapezoidal [13,15,16,18], or rectangular [8,9,10,11,12,14,17,19,20], and caused by the magnitude of the Deans number, Reynolds number, and the hydraulic diameter. Additional strategies include a more active approach on particle separation or particle movement in curved channels, such as dielectrophorsis [21,22] and magnetophoresis [23].

Recent experimental [24,25], theoretical [26], and numerical [27,28,29,30,31] studies, however, have validated a non-traditional strategy to manipulate the dynamics of non-spherical magnetic microparticles by coupling uniform magnetic fields and shear flows in straight channels. By applying a uniform magnetic field, the magnetic force is zero, but there exists a magnetic torque. The lateral migration of non-spherical particles relies on the coupling of the magnetic field, flow field, and particle-wall hydrodynamic interactions. With the understanding of the physics involved, Zhou et al. successfully separated ellipsoidal and spherical paramagnetic microparticles [24,25]. In the absence of a magnetic field, the ellipsoidal particle oscillates towards and away from the wall but results in a zero-net migration due to its symmetric rotation. In the presence of a magnetic field applied at an arbitrary direction, the symmetry of rotation is broken and the ellipsoidal particle has a net lateral migration towards or away from the wall. The migratory behavior is based on an oscillatory motion (weak magnetic regime) or a non-oscillatory motion as the ellipsoidal particle is pinned at a stable steady angle (strong magnetic regime). For a paramagnetic spherical particle, the magnetic torque is zero and its rotation cannot be manipulated by the uniform magnetic field [26]. Therefore, a spherical particle behaves the same with or without a magnetic field and its net migration remains zero. Additionally, the Reynolds number is less than one, thus making the lateral migration of a non-spherical particle highly dependent on its rotational behavior. As a result, when an ellipsoidal and a spherical particle are immersed in a low Reynolds number fluid flow in a straight channel and under a uniform magnetic field, it is feasible to separate particles by shape rather than relying on magnetic forces (where the strength rapidly decreases further away from the magnetic source), and inertial focusing (relies on the cross-sectional vortices and equilibrium positions for different shaped and sized particles).

Although recent experimental studies provide a useful strategy to separate particles by shape, it is still difficult to manage a well-controlled experiment, especially for curved channels. If we want to conduct a particle-focused experiment, the difficulty of studying a single particle in a curved channel is based on the many turns of a serpentine channel, gradual increasing radii of spiral channels, and large radii of a single curved channel. Additionally, it is difficult to observe the particle position in the cross-section of the channel. In other words, the difficulty of a curved channel experiment is based on optics.

On the other hand, numerical simulations are used as powerful tools to observe the transportation, rotation, and radial migration behavior of a particle. The particle dynamics are dependent on many parameters including the size and shape of the particle, the height, width, and shape of the channel cross-sections, low fluid velocities, radii of the curved channel, and the strength and direction of the magnetic field. For example, Harding et al. conducted a numerical and experimental study on a spherical particle transporting in a low Reynolds number flow in a curved channel by investigating the size of the particle and the cross-sectional shape, radii, width, and height of the channel [32,33]. In the experiments given, the larger particles migrated towards the inner channel wall and the results of the numerical study, conducted by Harding and Betozzi, concurred with the experimental findings [32]. In their article, however, their Reynolds number is large (greater than 50), whereas our Reynolds number in this paper is less than one and our particle is non-spherical.

Even though there are no published articles for non-spherical particles in curved channels and under uniform magnetic fields, it is an important subject for science and engineering regarding theoretical, experimental, and numerical analyses. The popular and revolutionary work of G.B. Jeffery investigated the simple shear flow acting on the particle in the absence of a channel wall [34] and the application of a uniform magnetic field, carried out by Zhou et al., has become recognizable for the separation of particles. Today, there have been many advancements towards computer simulations to study non-spherical particles in shear flows, Poiseuille flows, and under a uniform magnetic field. Some of the recent and successful numerical simulations, to study particles in Poiseuille and Couette flows and under a uniform magnetic field, were computed by Zhang et al. and Cao et al. by applying the arbitrary Lagrangian-Eulerian (ALE) algorithm for the finite element methods (FEM) and by using the direct numerical simulation (DNS) [27,30,31]. Further simulations from this method were successfully compared with the experimental and theoretical results of a neutrally buoyant particle under a uniform magnetic field [25,26,30].

In this paper, we focus on a two-dimensional study for an elliptical particle in low Reynolds number, Poiseuille flows in a curved channel, and under a uniform magnetic field. Therefore, the elliptical particle becomes dependent on the wall lift and the shear lift forces (hydrodynamic force), and the magnetic and hydrodynamic torques (to study its orientation). In the hydrodynamic section, we analyze the dynamics of the particle in two ways: one for a π periodic rotation and the other discussing the transportation throughout the upper half of the curved channel. Due to the hydrodynamic torque and force, our numerical simulations demonstrate the rotation and migration behavior of the elliptical particle. For its rotational behavior, we observe whether or not the particle executes a symmetric rotation, and we study its angular velocity. By analyzing a single periodic rotation, we examine the particle’s net radial migration and conclude the radial migration at the exit of the curved channel. We also analyze how different parameters affect the particle dynamics including: the particle aspect ratio, radii of the channel walls, and initial positions. Finally, we evaluate one of these parameters to show how the uniform magnetic field strength and direction affect the particle dynamics along with the aspect ratio of the particle, initial particle-wall distances, and the channel geometry. By applying a uniform magnetic field, we can examine the symmetrical property of the particle’s angular velocity, the oscillatory motion of the particle, and its average radial velocity. We apply a DNS by using a FEM, based on an ALE approach to analyze the coupling of the magnetic and the flow field, and to solve the dynamics and the transportation of the particle and the flow field (affected by the particle dynamics) in a curved channel. The hydrodynamic force and the magnetic and hydrodynamic toques are computed by a COMSOL FEM solver (5.2a, COMSOL Inc., Burlington, MA, USA) to find the orientation and the radial displacement motions by Newton’s second law of physics and Euler’s laws of motion.

## 2. Materials and Methods

### 2.1. Simulation Method and Mathematical Models

**Figure 1:**
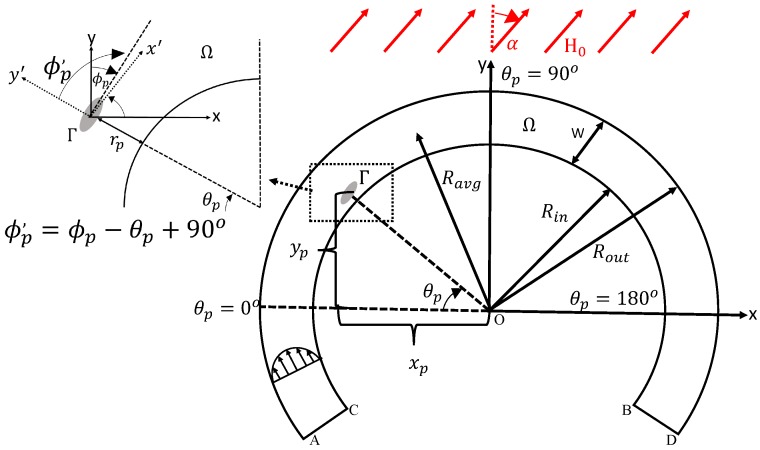
Schematic view of the numerical model of an elliptical particle that is neutrally buoyant in a Poiseuille flow and under a uniform magnetic field with strength $H_{0}$ and applied at direction α. The fluid and particle domains are Ω and Γ, respectively. The orientation of the particle is denoted as $\phi_{p}^{\prime }=\phi_{p}-\theta_{p}+90^{\circ }$ such that the laboratory frame is the $x^{\prime }-y^{\prime }$ axes, and $y^{\prime }$ is perpendicular to the channel wall, where $\phi_{p}$ is the global direction of the particle (x−y axes), and $\theta_{p}$ is the particle position inside the channel with respect to the origin of both curves, O. The particle-wall separation distance is denoted by $r_{p}$, and the channel geometry of the curve is denoted by the average radius $R_{avg}=\frac{R_{in}+R_{out}}{2}$, where $R_{in}$ and $R_{out}$ are the inner and outer radii, respectively, and *W* is the channel width.

We place a neutrally buoyant prolate elliptical particle in a Poiseuille flow of an incompressible Newtonian fluid, with density $\rho_{f}$ and dynamic viscosity $\eta_{f}$, as observed in Figure 1. The computational domain consists of a particle domain Γ and a fluid domain Ω enclosed by the boundary ABCD. The computational domain is bounded in a curved channel with an average radius $R_{avg}=\frac{R_{in}+R_{out}}{2}$ where the inner and outer radii of the walls are $R_{in}$ and $R_{out}$, respectively, and *W* is the width of the channel. The particle has an aspect ratio AR=a/b where *a* and *b* are the major and minor semi-axes lengths of the particle, respectively. A uniform magnetic field strength, $H_{0}$, is applied at a direction α. The particle-wall separation distance, $r_{p}$, is defined as the difference between the resultant length of the particle center of mass ($x_{p},y_{p}$) from the origin, O, and $R_{in}$. The particle position in the curved channel, $\theta_{p}$, is defined as the arc-tangent of the particle center of mass from the origin. In this study, $\theta_{p}=0^{\circ }$ indicates that the particle’s center of mass is at $-R_{out}<x_{p}<-R_{in}$ and $y_{p}=0$ μm (the channel entrance), $\theta_{p}=90^{\circ }$ indicates that $R_{in}<y_{p}<R_{out}$ and $x_{p}=0$ μm (halfway point), and $\theta_{p}=180^{\circ }$ indicates that $R_{in}<x_{p}<R_{out}$ and $y_{p}=0$ μm (the channel exit). The variable $\phi_{p}^{\prime }$, is defined as the lab frame angle of the particle between its major axis and the positive y’-axis. The y’-axis is perpendicular to the channel curve and is dependent on the particle $\theta_{p}$ position and the global frame of the particle orientation $\phi_{p}$ (the angle between its major axis and the positive y-axis):

(1) $$\begin{array}{c} \phi_{p}^{\prime }=\phi_{p}-\theta_{p}+90^{\circ }. \end{array}$$

The directions $\phi_{p}^{\prime }=0^{\circ }$ and $\phi_{p}^{\prime }=90^{\circ }$ indicate that the semi-major axis of the particle is perpendicular and parallel to the channel wall, respectively. In this study, we define the change in radial position as $\Delta r_{p\pi }=r_{p\pi }-r_{p0}$ as the difference between the particle-wall distance after one periodic rotation ($\phi_{p}^{\prime }$ rotates from $0^{\circ }$ to $180^{\circ }$). The variable $r_{p\pi }$ is the radial distance at the end of its periodic rotation $\phi_{p}^{\prime }=180^{\circ }$ and $r_{p0}$ is the initial radial position at the beginning of the periodic rotation $\phi_{p}^{\prime }=0^{\circ }$. We have another change in radial position as $\Delta r_{p\theta }=r_{p\theta }-r_{p0}$ where $r_{p\theta }$ is the radial position at the exit of the curved channel $\theta_{p}=180^{\circ }$ and $r_{p0}$ is the radial position at the entrance of the curved channel $\theta_{p}=0^{\circ }$. Therefore, in our simulations the initial radial position $r_{p0}$ happens at the orientation $\phi_{p}^{\prime }=0^{\circ }$ and at the position in the channel $\theta_{p}=0^{\circ }$. The particle either has a net radial migration towards the channel center, $\Delta r_{p\pi }>0$ μm ($\Delta r_{p\theta }>0$ μm), towards the channel wall, $\Delta r_{p\pi }<0$ μm ($\Delta r_{p\theta }<0$ μm), or neither, $\Delta r_{p\pi }=0$ μm ($\Delta r_{p\theta }=0$ μm).

The flow field, u, is governed by the continuity and the Navier–Stokes equations for an incompressible Newtonian flow with a no-slip condition on the channel walls AD and CB. The inlet of the channel is at AC and becomes a fully developed laminar flow, and BD is the outlet where the normal pressure is zero:

(2) $$\begin{array}{c} \;\nabla \cdot u=0, \end{array}$$

(3) $$\begin{array}{c} \rho_{f}\left[\frac{\partial u}{\partial t}+\left(u\cdot \nabla \right)u\right]=-\nabla p+\nabla \cdot \nu_{f}\left(\nabla u+{\left(\nabla u\right)}^{T}\right), \end{array}$$

where *p* is the pressure and *t* is time.

Since there is also a no-slip condition on the surface of the elliptical particle, the fluid velocity on the the particle surface is a function of the particle angular and translation velocities:

(4) $$\begin{array}{c} u=U_{p}+\omega_{p}\times \left(x_{s}-x_{p}\right), \end{array}$$

where $U_{p}$ and $\omega_{p}$ are the translational and rotational velocities of the particle, respectively, and $x_{s}$ and $x_{p}$ are the vector positions of the particle surface and center of mass.

The hydrodynamic force and torque acting on the particle are also given:

(5) $$\begin{array}{c} F_{h}=\int \left(\tau_{h}\cdot n\right)dS, \end{array}$$

(6) $$\begin{array}{c} T_{h}=\int \left(\tau_{h}\times \left(x_{s}-x_{p}\right)\cdot n\right)dS, \end{array}$$

where $\tau_{h}=\eta_{f}\left(\nabla u+{\left(\nabla u\right)}^{T}\right)$ is the hydrodynamic stress tensor. The governing equations of the magnetic field are given from Maxwell’s equations:

(7) $$\begin{array}{c} \nabla \times H=0, \end{array}$$

(8) $$\begin{array}{c} \nabla \cdot B=0, \end{array}$$

where H and B are the magnetic field strength and flux density, respectively. It should be mentioned that the fluid is non-magnetic and the channel walls, AD and CB, are magnetically insulated. When a uniform magnetic field is applied onto a particle, the magnetic torque is non-zero but the magnetic force is negligible. When calculating the magnetic torque, we assume that the paramagnetic particle is homogeneous, isotropic, and linearly magnetizable:

(9) $$\begin{array}{c} T_{m}=\mu_{0}V_{p}\chi_{p}H^{-}\times H_{0}, \end{array}$$

where $\mu_{0}$ is the magnetic permeability of free space, $\chi_{p}$ is the paramagnetic particle magnetic susceptibility, $V_{p}$ is the particle volume, and the magnetic field strengths are inside, $H^{-}$, and outside, $H_{0}$, of the particle.

For the hydrodynamic force and the hydrodynamic and magnetic torques acting on the particle, we have a set of equations:

(10) $$\begin{array}{c} m_{p}\frac{dU_{p}}{dt}=F_{h}, \end{array}$$

(11) $$\begin{array}{c} I_{p}\frac{d\omega_{p}}{dt}=T_{h}+T_{m}, \end{array}$$

where $m_{p}$ and $I_{p}$ are the mass and the moment of inertia of the particle. The time-dependent position of the particle center of mass $C_{p}\left(t\right)=\left(x_{p},y_{p}\right)$ and orientation $\phi_{p}$ are calculated by:

(12) $$\begin{array}{c} C_{p}\left(t\right)=C_{p}\left(0\right)+\int_{0}^{t}U_{p}\left(s\right)ds, \end{array}$$

(13) $$\begin{array}{c} \phi_{p}\left(t\right)=\phi_{p}\left(0\right)+\int_{0}^{t}\omega_{p}\left(s\right)ds, \end{array}$$

where $C_{p}\left(0\right)$ and $\phi_{p}\left(0\right)$ are the initial position and orientation of the particle.

The rotation and radial migration of the paramagnetic particle are affected by the hydrodynamic force and the coupling of hydrodynamic and magnetic torques. Likewise, every channel, radial, and orientation positions of the particle will cause a change in the magnetic and hydrodynamic torques and the fluid flow. Given the geometry of the channel and the calculations that include the radial position and orientation of the particle, we use direct numerical simulation (DNS) from the finite element method (FEM), and an arbitrary Lagrangian-Eulerian (ALE) method for the coupling on the particle, fluid flow, and the uniform magnetic field [27,30]. Our simulations were solved by numerical modeling using a commercial FEM solver COMSOL Multiphysics. Similar to previous articles, we use a stationary solver for parametric sweep analysis to simulate the magnetic field inside and outside of the particle, and calculate the magnetic torque on the particle [27,30]. We then apply a time-dependent solver for a particle-fluid interaction model, and we imported a variable that represents the magnetic torque. We use a piecewise function to activate the uniform magnetic field and the magnetic torque on the particle at a time, t, when $\theta_{p}\approx 0^{\circ }$. To estimate an accurate calculation on the particle surface by the torques and the hydrodynamic force, we use a fine quadratic triangular mesh around the particle and a finer quadratic triangular mesh at the tip of the particle.

The accuracy and convergence of our simulation is based on the number of elements in the computational domain Ω, particle surface Γ, and the time step Δt. As part of our numerical simulation setup in Figure 1, we use 18,571 domain elements for Ω, and 152 boundary elements for Γ. The number of elements establishes time efficient calculations, while also ensuring accurate results of the particle transportation and rotation. For the fluid mechanics acting on the particle surface, we use the time-step function to have the Poiseuille flow to reach its average velocity and become fully developed before the particle approaches $\theta_{p}=0^{\circ }$ with a time step $\Delta t=1\times 10^{-5}$ s. When the particle position is $\theta_{p}\approx 0^{\circ }$, we set $\phi_{p0}^{\prime }\approx 0^{\circ }$ since the semi-major axis of the particle is almost perpendicular to the channel wall. Additionally, we start our new initial radial position, $r_{p0}$, and our initial time at t=0 s. Comparisons between the computational meshes for time step $\Delta t=1\times 10^{-5}$ s and comparisons between time steps for 18,571 domain elements and 152 boundary elements can be seen in Appendix B.

### 2.2. Material Properties

In the following computed simulations, the fluid property is a water-based material with a density of 1000 kg/m${}^{3}$ and the dynamic viscosity is $1.002\times 10^{-3}$ Pa·s, the width of the channel is kept at 50 μm, and the inlet flow velocity is $U_{avg}=2.5$ mm/s. The Reynolds number is defined as $R_{e}=\frac{\rho_{f}U_{avg}W}{\eta_{f}}$, and the value in the simulation setup is $R_{e}=0.125$, thus placing the elliptical particle in a laminar flow where the fluid and particle inertia are small. Due to the low Reynolds number, we assume that any secondary flow is neglected for a two-dimensional study on a particle. The particle is a magnetic-doped polystyrene particle with a magnetic susceptibility of $\chi_{p}=0.26$, and the fluid is non-magnetic. For some parts of our analysis, we will analyze elliptical particles with various aspect ratios, but they will have the same volume as a 7 μm-diameter circular particle.

## 3. Results and Discussion

### 3.1. Particle Dynamics in a Uniform Magnetic Field

In this section, we investigate the transportation and rotation of an elliptical particle in the presence of a uniform magnetic field near the inner wall whereas an elliptical particle near the outer wall is discussed in Appendix A. We keep AR=4, $r_{p0}=12$ μm, $R_{avg}=175$ μm, and the magnetic field is applied at either $\alpha =0^{\circ }$ or $\alpha =90^{\circ }$. In our numerical simulation, we activate the magnetic field by using a piecewise function at a time when $\theta_{p}\approx 0^{\circ }$. At this point, we establish our new initials ($\phi_{p0}^{\prime }$ and $r_{p0}$) and set t=0 s. We have compared some of our results to an elliptical particle in the absence of a magnetic field observed in Appendix C.

#### 3.1.1. Magnetic Field at α = 0${}^{\circ }$

**Figure 2:**
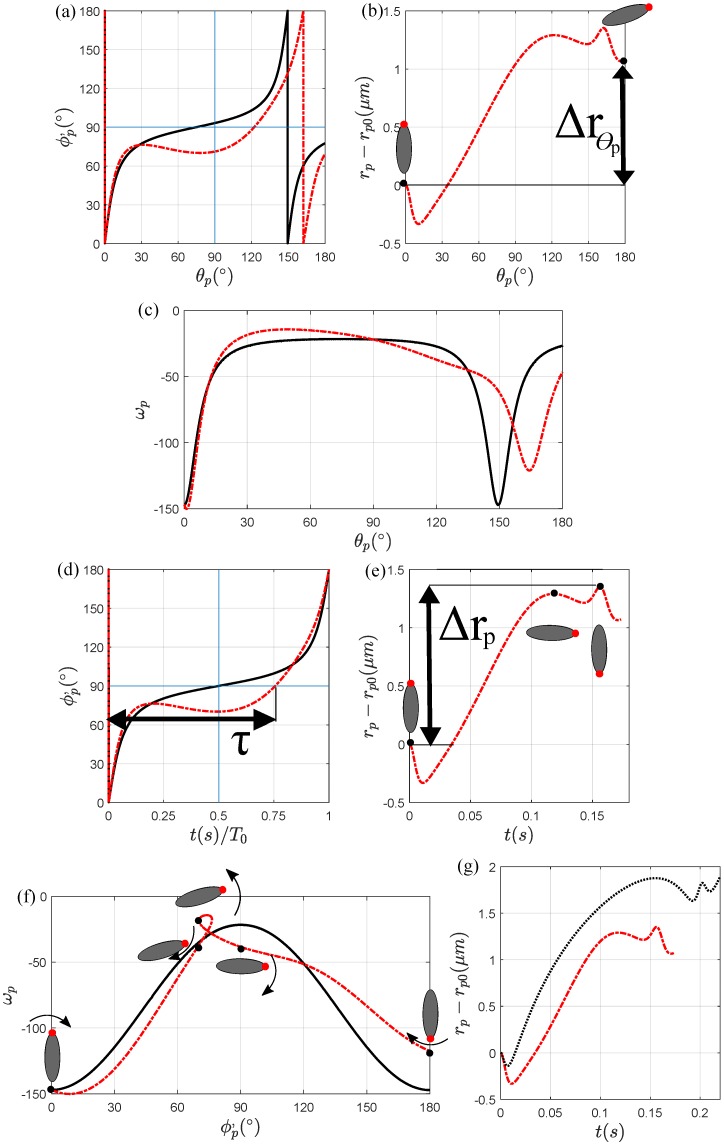
Particle dynamic and transportation comparison between $H_{0}=0$ A/m (solid black line), and $H_{0}=3000$ A/m applied at $\alpha =0^{\circ }$ (dot-dash red line) for (**a**) the particle’s rotation, (**b**) the radial migration, and (**c**) the angular velocity in terms of $\theta_{p}$; (**d**) the particle’s rotation, (**e**) the radial migration, (**f**) the angular velocity in terms of time *t*, and (**g**) comparison between the particle-wall distances in a straight channel (dot black line) and in a curved channel (dot-dash red line).

In this section, we apply different magnetic field strengths at $\alpha =0^{\circ }$ to study the particle rotation and the particle-wall distance as the particle is transporting in a curved channel. Figure 2a,b, each exhibiting the particle’s rotation and radial migration, respectively, as a function of $\theta_{p}$, shows the comparison between them and $H_{0}=0$ A/m and $H_{0}=3000$ A/m. For both magnetic field strengths, we observe where in the channel the particle completes the first half of its rotation. At $H_{0}=0$ A/m, the first half rotation of a particle ($\phi_{p}^{\prime }=90^{\circ }$) occurs in the first half of the curved channel ($\theta_{p}=74^{\circ }$). On the other hand, when $\alpha =0^{\circ }$ and $H_{0}=3000$ A/m, the particle approaches the first half of its rotation in the second half of the curve ($\theta_{p}=122^{\circ }$), indicating that the magnetic field affects the orientation of the particle during its transportation. We reintroduce the dimensionless variable τ as a function of the particle aspect ratio, radii and shape of the curve geometry, initial particle-wall distance, direction of the particle, and the direction and strength of the magnetic field. We see that in Figure 2d, a particle in the absence of a magnetic field executes symmetric rotations (τ=0.50), while a particle in a magnetic field experiences an asymmetric rotation (τ=0.76). We use the rotation of the particle to analyze the net radial migration of the particle. Due to the asymmetric rotation, the particle radially migrates toward the channel center after one periodic rotation ($\Delta r_{p\pi }>0$ μm), seen in Figure 2e, and at the channel exit ($\Delta r_{p\theta }>0$ μm), seen in Figure 2b, for the magnetic field strength $H_{0}=3000$ A/m applied at $\alpha =0^{\circ }$.

The observation of an elliptical particle’s dynamics under a magnetic field applied at $\alpha =0^{\circ }$ can thus be explained. In the absence of a magnetic field, the particle rotates due to the hydrodynamic torque. The particle, however, has a net zero migration after one periodic rotation, but migrates towards the channel wall at $\theta_{p}=180^{\circ }$, as shown in Appendix C. In Figure 2a, we see that the applied magnetic field strength and direction rotates the particle backwards towards $\phi_{p}^{\prime }=0^{\circ }$ due to the direction and magnitude of the shear rate at the particle position inside the channel and its distance from the wall. In the range $31^{\circ }\leq \theta_{p}\leq 78^{\circ }$, the particle exposed to $H_{0}=3000$ A/m rotates toward $\phi_{p}^{\prime }=0^{\circ }$ because the magnetic torque is stronger than the hydrodynamic torque (i.e., the particle is in a strong field regime) and the orientation of the particle at that $\theta_{p}$ range approaches towards its stable steady angle [25,26,27,30]. The rotation of the particle over time is thus caused by the hydrodynamic and magnetic angular velocities, shown in Figure 2c,f. The magnetic angular velocity, $\omega_{m}$, is in the opposite direction of the hydrodynamic angular velocity, $\omega_{h}$, in the first half of the particle orientation $0^{\circ }<\phi_{p}^{\prime }<90^{\circ }$ and is in the same direction for $90^{\circ }<\phi_{p}^{\prime }<180^{\circ }$. As a result, the particle will spend a longer time in the first half of its rotation compared to its second half (i.e., the particle rotation becomes more asymmetric). Thus, at a certain position $\theta_{p}$, the particle orientation allows the particle to radially migrate away from the wall faster due to the rotational dynamics affected by the magnetic field and the wall lift force caused by the hydrodynamic interaction. Otherwise, the particle continues its periodic rotation in other parts of the channel since the magnetic field is either considered weak compared to the hydrodynamic angular velocity or both angular velocities are in the same direction.

**Figure A5:**
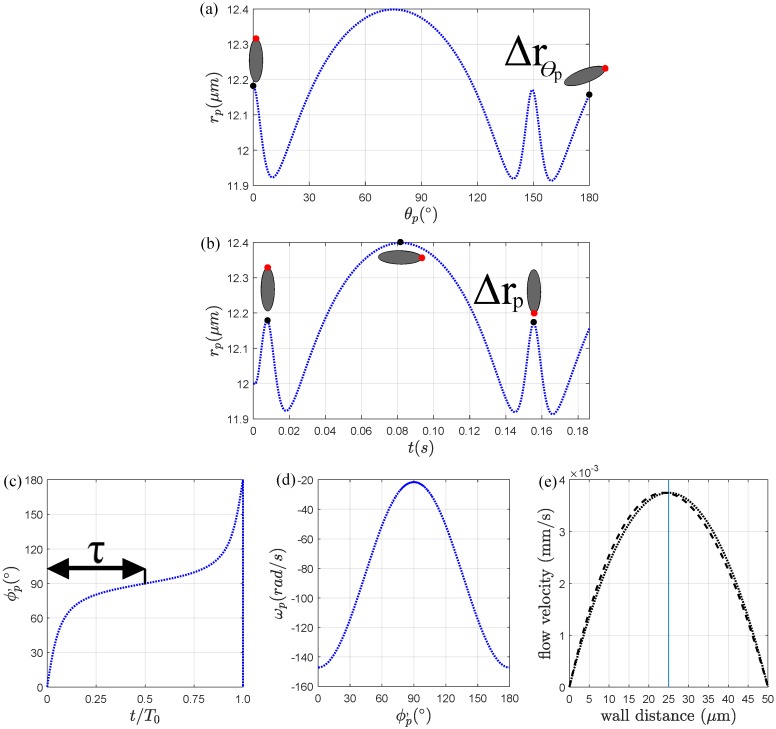
Translation and rotation of an elliptical particle in the absence of a magnetic field. The particle has an aspect ratio AR=4 and the initial radial position is $r_{p0}=12$ μm, whereas the initial particle direction is $\phi_{p0}=43^{\circ }$ in the beginning of the simulation. The channel’s average radius is $R_{avg}=175$ μm. The particle-wall distance is studied with respect to its (**a**) $\theta_{p}$ position; (**b**) time, t, in the curved channel; (**c**) the evolution of the orientation angle $\phi_{p}^{\prime }$ with respect to dimensionless time $t/T_{0}$; (**d**) the rotational velocity $\omega_{p}$ versus $\phi_{p}^{\prime }$; and (**e**) the velocity profiles for a curved channel (dashed line) and straight channel (dotted line).

The result of the particle net radial migration after one periodic rotation and at the exit of the curved channel can be seen in Figure 2b,e. Compared to a particle in the absence of a magnetic field, the particle exposed to a magnetic field strength $H_{0}=3000$ A/m migrates toward the channel center, $\Delta r_{p\pi }>0$ μm. In this case, the oscillatory migration is positive for both ranges $0^{\circ }<\phi_{p}^{\prime }<90^{\circ }$ and $90^{\circ }<\phi_{p}^{\prime }<180^{\circ }$. For the particle transportation throughout the channel curve under a uniform magnetic field, the net radial migration results in $\Delta r_{p\theta }>0$ μm compared to a particle in the absence of a magnetic field in Appendix C, Figure A5.

To further understand the particle angular velocity and its net radial migration, we must also understand its time-dependent periodic rotation and its rotation during the transportation throughout the curved channel. Figure 2d,e shows the particle rotation and radial migration with respect to dimensionless and dimensional time, respectively. In Figure 2d, when $H_{0}=0$ A/m, τ=0.50 ($T_{0}=0.15$ s) and is almost symmetric, whereas the magnetic field strength $H_{0}=3000$ A/m allows τ=0.76 ($T_{0}=0.16$ s), thus making the particle rotation more asymmetric. In Figure 2e, we see that increasing τ will also increase the net radial migration towards the channel center due to the magnetic field and the magnitude of the shear rate at the particle-wall distance. Since the shear rate decreases closer to the channel center, and the oscillatory motion is caused by the coupling between the magnetic field and the shear rate, a greater asymmetric rotation causes the particle to radially migrate further. Therefore, by adding the magnetic field at $\alpha =0^{\circ }$, the rotation affected by $T_{0}$, and τ results in an increase in the particle-wall distance.

The magnetic field’s influence on the particle’s rotation and radial migration is based on the magnetic and hydrodynamic torques, and consequentially, their angular velocities. We see in Figure 2f that the total particle angular velocity $\omega_{p}=\omega_{h}+\omega_{m}$ is a function of the particle direction $\phi_{p}^{\prime }$ for one periodic rotation in the curved channel. When the magnetic field is applied at $\alpha =0^{\circ }$, there exists a ’loop’ for the orientation $70^{\circ }<\phi_{p}^{\prime }<76^{\circ }$ in the range $31^{\circ }\leq \theta_{p}\leq 78^{\circ }$. In this case, the particle rotates backward because the magnetic torque is changing directions, as well as the hydrodynamic torque. By considering the particle channel position $\theta_{p}$, its radial position $r_{p}$, and its orientation $\phi_{p}^{\prime }$, the magnetic angular velocity is greater than the hydrodynamic angular velocity. In Figure 2c, we compare the angular velocities between a particle with and without a magnetic field during its transportation. While a particle is executing is periodic rotation, the angular velocity profile is symmetric in the absence of a magnetic field. After the periodic rotation, the particle will continue to execute symmetric rotations due to the shear rate at the particle-wall distance. In the presence of a magnetic field, however, the angular velocity is largely asymmetric and the angular velocity at $\theta_{p}=162.2^{\circ }$ (when $\phi_{p}^{\prime }=180^{\circ }$) has increased. Thus, the disproportionate angular velocity and the asymmetric periodic rotation contributes to the particle net radial migration.

We finally compare the particle-wall distances between a particle in a straight channel and a particle in a curved channel, as seen in Figure 2g. In both simulations, all conditions are the same except one channel is curved, while the other channel is straight. As we can see, we expect different results between a particle in a straight channel and a particle in a curved channel because $\theta_{p}$ is considered constant for a straight channel ($\theta_{p}=90^{\circ }$), whereas $\theta_{p}$ constantly changes throughout the curved channel. Therefore, since the magnetic torque constantly changes directions in a curved channel, the particle’s rotational dynamics will be affected and, consequentially, so will its particle-wall distance. We notice that after one periodic rotate, both particles will migrate away from the wall, but a particle in a straight channel will migrate faster from the wall than a particle in a curved channel.

**Figure 3:**
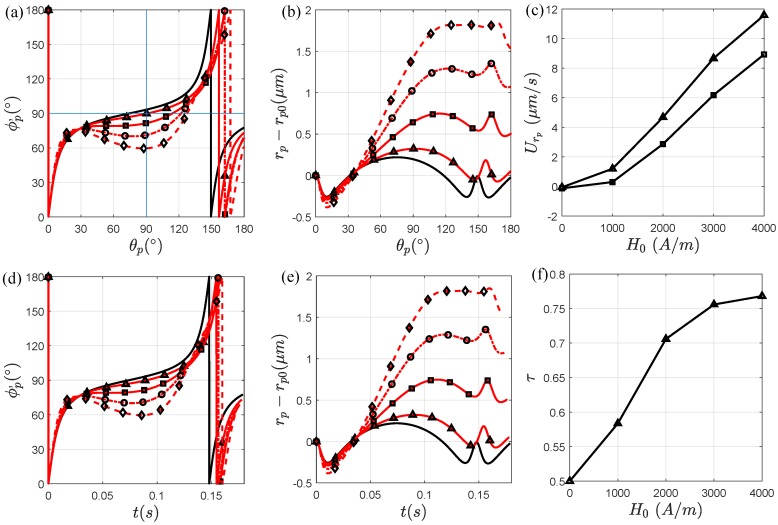
Effect of the magnetic field strength when it is applied at $\alpha =0^{\circ }$. The particle (AR=4) is affected by the magnetic field when its center of mass is approximately at $\theta_{p}=0^{\circ }$ in (**a**,**d**) when its rotation is with respect to $\theta_{p}$ and time, respectively, for, $H_{0}=0$ A/m (solid black line), $H_{0}=1000$ A/m (triangle symbol), $H_{0}=2000$ A/m (square symbol), $H_{0}=3000$ A/m (circular symbol), and $H_{0}=4000$ A/m (diamond symbol). The radial particle-wall distance change of the particle with respect to (**b**) $\theta_{p}$, and (**e**) time. (**c**) The radial velocities of the particle $U_{rp}$ (triangle symbol) and $U_{r\theta }$ (square symbol) as functions of $H_{0}$. (**f**) The dimensionless parameter τ as a function of $H_{0}$. We see that as τ increases, the net migration of the particle increases.

Now that we have established how the particle rotation and migration is affected by applying a magnetic field, we analyze how increasing the magnetic field strength affects the orientation and radial migration of an elliptical particle. Figure 3a,d shows the orientation of the particle, $\phi_{p}^{\prime }$, with respect to $\theta_{p}$ and time t, respectively. In Figure 3a, as the magnetic field strength increases from $H_{0}=1000$ A/m to $H_{0}=4000$ A/m, the rotation of the particle becomes more asymmetric, and the magnetic torque and angular velocity becomes more dominant for a wider range of $\theta_{p}$. For a magnetic field strength $H_{0}=2000$ A/m, a backward orientation occurs in the range $52^{\circ }\leq \theta_{p}\leq 61^{\circ }$, $31^{\circ }\leq \theta_{p}\leq 78^{\circ }$ for $H_{0}=3000$ A/m, and $24^{\circ }\leq \theta_{p}\leq 88^{\circ }$ for $H_{0}=4000$ A/m. The particle is therefore exposed to a strong field regime for a larger portion of the curved channel. In Figure 3d, the periodic rotation time, $T_{0}$, increases. For an increasing magnetic field strength, the periodic rotation times are $T_{0}=0.147$ s for $H_{0}=0$ A/m, $T_{0}=0.154$ s for $H_{0}=1000$ A/m, $T_{0}=0.157$ s for $H_{0}=2000$ A/m, $T_{0}=0.156$ s for $H_{0}=3000$ A/m, $T_{0}=0.160$ s for $H_{0}=4000$ A/m. Consequentially, τ increases in (f) where the orientation becomes more asymmetrical. As a result, for an increasing magnetic field strength, both $\Delta r_{p\pi }$ and $\Delta r_{p\theta }$ increase as seen in Figure 3b,d, respectively. Similar to previous studies, we study the particle net radial migration by calculating the average radial migration velocities, $U_{r_{p\pi }}=\Delta r_{p\pi }/t$ and $U_{r_{p\theta }}=\Delta r_{p\theta }/t$, where *t* is replaced with $T_{0}$ or the time that it takes for the particle to transport through the channel [27,30]. The average radial velocities for different magnetic fields strengths are shown in Figure 3c for $U_{r_{p\pi }}$ (triangle symbol) and $U_{r_{p\theta }}$ (square symbol). We see that both radial velocities increase as the magnetic field strength increases, suggesting that the particle migrates faster from the inner wall due to the angular oscillatory motion in lower shear rates.

The rotation and migration behavior of an elliptical particle exposed to a magnetic field applied at $\alpha =0^{\circ }$ can be explained as follows. While a particle is traveling in the curved channel, the shear rate is constantly changing directions at every $\theta_{p}$. Therefore, if we apply a magnetic field at $\alpha =0^{\circ }$, the magnetic torque acting on the particle also changes its direction and the particle has a different rotation and migration experience during its transportation. For example, when the position of the particle is at $\theta_{p}=90^{\circ }$, it experiences a magnetic torque where the magnetic field is applied at $\alpha =0^{\circ }$ because the magnetic torque is perpendicular to the shear flow. On the other hand, a particle at $\theta_{p}=0^{\circ }$ experiences a magnetic torque similar to the magnetic field applied at $\alpha =90^{\circ }$ because the magnetic torque is parallel to the shear flow. Therefore, in some ranges of $\theta_{p}$, the particle experiences a backwards orientation because the particle is considered to be inside a strong magnetic field regime [25,26,27,30]. In all other regions, however, the particle is either in a weak field regime or the magnetic and hydrodynamic angular velocities are in the same direction. Thus, the effect on the particle orientation depends on the direction of the hydrodynamic and magnetic torques and the strength and direction of the magnetic field. It should also be mentioned that the particle orientation is influenced by its radial position; its orientation can be easily influenced if the radial position of the particle is in a low shear rate (closer to the channel center) versus a high shear rate (closer to the channel wall). After a full periodic rotation, some magnetic field strengths allows the particle to oscillate away from the wall due the channel geometry, the orientation of the particle, and the shear flow direction. Thus, by controlling the orientation of the particle, we also control its net radial migration and $\Delta r_{p\theta }>0$ μm.

#### 3.1.2. Magnetic Field at $\alpha =90^{\circ }$

**Figure 4:**
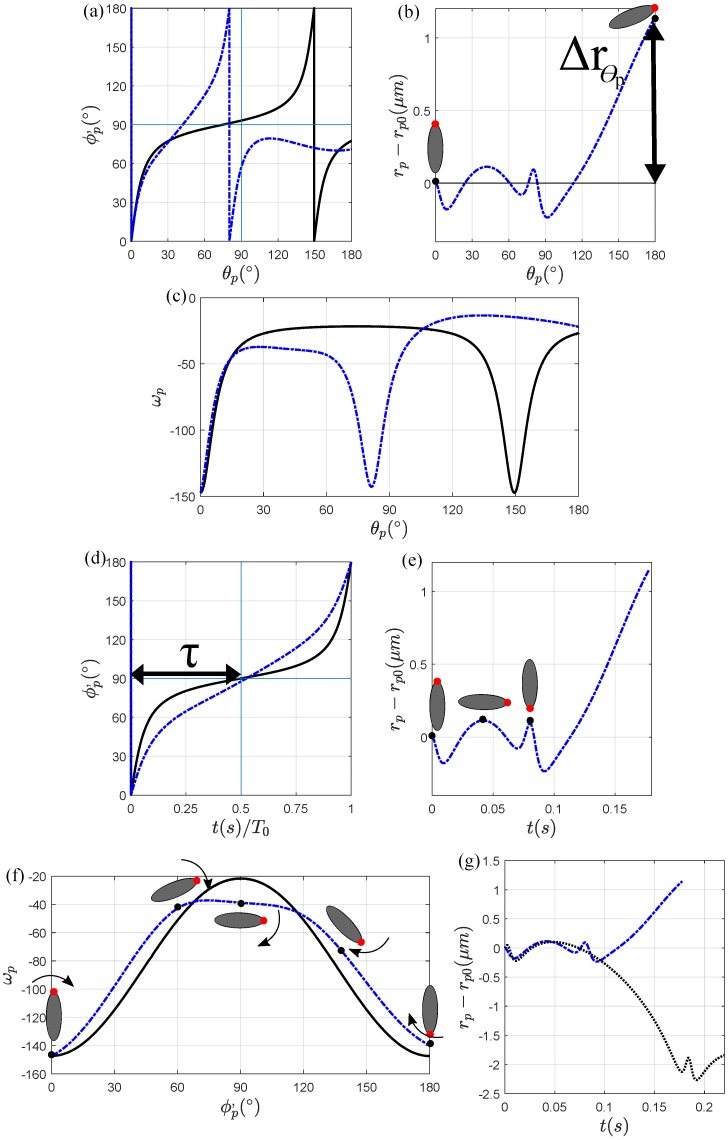
Particle dynamic and transportation comparison between $H_{0}=0$ A/m (solid black line), and $H_{0}=3000$ A/m applied at $\alpha =90^{\circ }$ (dot-dash blue line) for (**a**) the particle’s rotation, (**b**) the radial migration, and (**c**) the angular velocity in terms of $\theta_{p}$; (**d**) the particle’s rotation, (**e**) the radial migration, (**f**) the angular velocity in terms of time *t*, and (**g**) comparison between the particle-wall distances in a straight channel (dot black line) and in a curved channel (dot-dash blue line).

In this section, we apply a magnetic field at $\alpha =90^{\circ }$ to study the particle dynamics in a curved channel. Figure 4a,b, showing the particle orientation and radial migration, respectively, as a function of $\theta_{p}$, compares them with the magnetic field strengths $H_{0}=0$ A/m and $H_{0}=3000$ A/m. The first half rotation of the particle, $\phi_{p}^{\prime }=90^{\circ }$, occurs at $\theta_{p}=74^{\circ }$ for $H_{0}=0$ A/m and at $\theta_{p}=42^{\circ }$ for $H_{0}=3000$ A/m. One periodic rotation of the particle results in a positive net radial migration, as shown in Figure 4e. Similar to the magnetic field at $\alpha =0^{\circ }$, Figure 4d shows that, in the absence of a magnetic field, a particle executes a symmetric rotation (τ=0.50), while a particle exposed to a magnetic field strength of $H_{0}=3000$ A/m experiences a slight asymmetric rotation (τ=0.53). The reason for the particle periodic rotation and radial migration can be explained in a way similar to our analysis of $\alpha =0^{\circ }$. We see that in Figure 4f, for a range of $\theta_{p}$, the magnetic angular velocity, $\omega_{m}$, and the hydrodynamic angular velocity, $\omega_{h}$, are in an opposite direction in the first half of the particle orientation and performs in the same direction in the second half of its orientation. We also notice that unlike $\alpha =0^{\circ }$, there does not exist a ’loop’ during its periodic rotation even though the orientation is asymmetric with respect to $\phi_{p}=90^{\circ }$. For this range of $\theta_{p}$, the particle will be considered to be in a weak field regime, but the particle spends more time in the first half of its rotation and therefore results in a positive radial migration.

The large net radial migration towards the channel center as the particle exits the curved channel, $\Delta r_{p\theta }>0$ μm, can be explained by the particle transportation. Even though $\Delta r_{p\pi }=0$ μm and $\Delta r_{p\theta }<0$ μm in the absence of the magnetic field, we observe that the particle in a magnetic field strength of $H_{0}=3000$ A/m and at a direction of $90^{\circ }$ behaves similarly as a particle exposed to a magnetic field strength of $H_{0}=3000$ A/m applied at $\alpha =0^{\circ }$. In Figure 4a, we see that the magnetic field strength of $H_{0}=3000$ A/m applied at $\alpha =90^{\circ }$ does not result in a backwards orientation in the same region as $\alpha =0^{\circ }$ due to the direction and magnitude of the shear rate in the range $31^{\circ }<\theta_{p}<78^{\circ }$, $r_{p}$, and the strength and direction of the magnetic field. The particle, however, rotates backwards in the range $113^{\circ }<\theta_{p}<168^{\circ }$ in Figure 4a,c. Compared to $\alpha =0^{\circ }$, the region where the particle ends up in a backwards orientation increases by $81^{\circ }<\theta_{p}<91^{\circ }$. In Figure 4c, we compare the angular velocities between a particle with and without a magnetic field as it is transporting inside the curved channel. The angular velocity profile is symmetric in the absence of a magnetic field, whereas a magnetic field causes the angular velocity to become asymmetric in a magnetic field strength of $H_{0}=3000$ A/m applied at $\alpha =90^{\circ }$. For a certain position $\theta_{p}$, the particle orientation $\phi_{p}^{\prime }$ allows a positive net radial migration away from the wall faster due to the rotational dynamics affected by the magnetic field and the wall lift force. The particle is able to execute a periodic rotation in most parts of the channel given that either the magnetic torque is considered to be weak compared to the hydrodynamic torque or the magnetic and hydrodynamic torque are in the same direction. The result of the net radial migration throughout the curved channel can be seen in Figure 4b. Compared to a particle in the absence of a magnetic field, the particle exposed to a magnetic field strength of $H_{0}=3000$ A/m will result in a positive radial migration away from the inner channel wall, $\Delta r_{p\theta }>0$ μm.

Similar to what we did in Section 3.1.1, we compare the particle-wall distance between a particle in a straight channel and a particle in a curved channel as seen in Figure 4g. We again see that since the magnetic torque constantly changes directions in a curved channel, the particle’s rotational dynamics will be affected and consequentially, so will its particle-wall distance. We see that after one periodic rotate, a particle in a straight channel will migrate towards the wall, whereas a particle in a curved channel will radially migrate away from the wall. Therefore, we can conclude that the particle-wall distance will be greatly affected not only by different magnetic field strengths and directions, but also in a different channel geometry. Additionally, for magnetic field directions $\alpha =0^{\circ }$ and $\alpha =90^{\circ }$, and for the magnetic field strength $H_{0}=3000$ A/m, a particle in a curved channel will complete its periodic rotation faster than in a straight channel, resulting in a short particle-wall distance change in a curved channel than in a straight channel.

**Figure 5:**
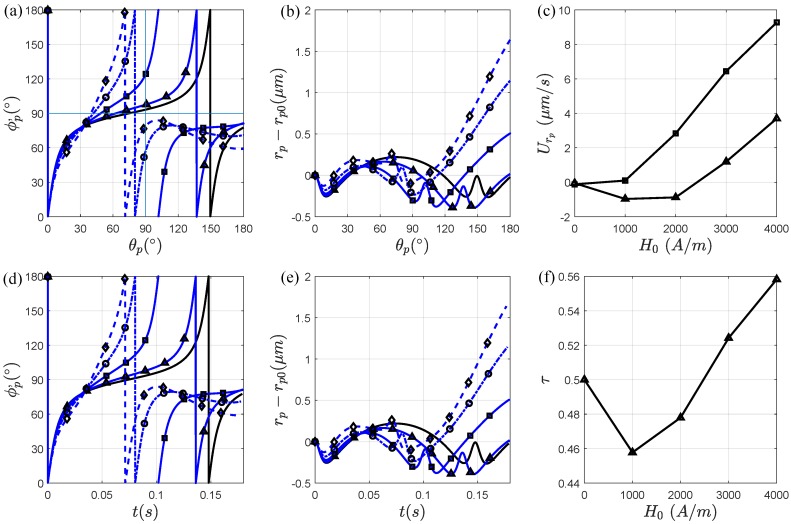
Effect of the magnetic field strength when it is applied at $\alpha =90^{\circ }$. The particle (AR=4) is affected by the magnetic field when its center of mass is approximately at $\theta_{p}=0^{\circ }$ in (**a**,**d**) when its rotation is with respect to $\theta_{p}$ and time, respectively, for, $H_{0}=0$ A/m (solid black line), $H_{0}=1000$ A/m (triangle symbol), $H_{0}=2000$ A/m (square symbol), $H_{0}=3000$ A/m (circular symbol), $H_{0}=4000$ A/m (diamond symbol). The radial particle-wall distance change of the particle with respect to (**b**) $\theta_{p}$, and (**e**) time. (**c**) The radial velocities of the particle $U_{rp}$ (triangle symbol) and $U_{r\theta }$ (square symbol) as functions of $H_{0}$. (**f**) The dimensionless parameter τ as a function of $H_{0}$. We see that as τ increases, the net migration of the particle increases.

Now that we have analyzed how a magnetic field applied at $\alpha =90^{\circ }$ affects the particle orientation and migration, we evaluate how increasing the magnetic field strength affects the orientation and net radial migration of the particle. Figure 5a,d shows the orientation of the particle, $\phi_{p}^{\prime }$, with respect to $\theta_{p}$ and time t, respectively. In Figure 5a, as the magnetic field strength increases from $H_{0}=1000$ A/m to $H_{0}=4000$ A/m the particle orientation becomes more asymmetric and the magnetic torque and angular velocity become more dominant for a wider range of $\theta_{p}$ near the channel exit. The particle is therefore exposed to a strong field regime for a large portion of the second half of the curved channel. In Figure 5d, the periodic rotation time $T_{0}$ decreases and, consequentially, τ increases in Figure 5f. The particle-wall distance also increases after one periodic rotation and upon exiting the channel curve in Figure 5b,e as a result of increasing $H_{0}$. In Figure 5c, the average radial velocities, $U_{r_{p\pi }}$ (triangle symbol) and $U_{r_{p\theta }}$ (square symbol), increase as the magnetic field strength increases, resulting in a fast radial migration away from the wall.

### 3.2. Effect Due to Changes in Parameters

By changing the aspect ratio, average radius, and/or initial position, the elliptical particle experiences a different orientation and net radial migration, as shown in Appendix C. For these parameters, we can explain what can occur in the presence of a magnetic field based on our observations for AR=4, $R_{avg}=175$ μm, and $r_{p0}=12$ μm in Section 3.1.1 and Section 3.1.2.

In the absence of a magnetic field, for larger aspect ratios, $T_{0}$ increases and the particle results in a zero net migration for all aspect ratios. Based on past analyses, for a magnetic field strength applied at $\alpha =0^{\circ }$ and $\alpha =90^{\circ }$, the smaller aspect ratio particles are more susceptible to the magnetic field for the same shear rate [25,26,27,30]. Therefore, if the magnetic torque and angular velocity influences on a similar particle is greater than the hydrodynamic torque and angular velocity (for a large range of $\theta_{p}$), then $T_{0}$ decreases and τ increases [25,26,27,30]. As a result, the particles radially migrates toward the channel center.

Next, when the aspect ratio is the same but the average radius increases, $T_{0}$ decreases, but $\Delta r_{p\theta }$ increases. When we apply a magnetic field, the value of τ results either in a net positive or negative radial migration and can change, depending on the average radius of the channel. Therefore, for an increasing $R_{avg}$, a particle with an aspect ratio AR=4 is more susceptible to the magnetic field and has a larger net migration. For smaller $R_{avg}$, the particle transportation is faster and $T_{0}$ increases and thus does not react to a uniform magnetic field. For a larger $R_{avg}$, the particle is more susceptible to the magnetic field because the particle spends more time in the curved channel and $T_{0}$ decreases. Even though $T_{0}$ decreased for an increasing $R_{avg}$, a magnetic field can manipulate the orientation of the particle and its net migration efficiently by increasing the magnetic field strength and τ.

Finally, as $r_{p0}$ increases, $T_{0}$ increases since the shear rate is lower towards the channel center. Therefore, it is obvious that since the magnetic field breaks the rotation of symmetry and increases $T_{0}$ and τ, the particle orientation is easily manipulated for a larger range of $\theta_{p}$. By controlling the orientation in the range $0^{\circ }<\phi_{p}^{\prime }<90^{\circ }$, the particle migrates toward the channel center or toward the inner channel wall in the range $90^{\circ }<\phi_{p}^{\prime }<180^{\circ }$.

## 4. Conclusions

We numerically investigated the orientation and radial migration of a paramagnetic elliptical particle in a Poiseuille flow in a curved channel and under a uniform magnetic field. We accomplished our numerical computations by using a multi-physics numerical model based on a direct numerical simulation and a finite element method that uses an arbitrary Lagrangian-Eulerian approach. Simulations were used to analyze how the geometry of the channel, particle-wall distance, shear rate, the shape of the particle, and the strength and direction of the magnetic field affect the orientation and net radial migration of the particle. The physics in this paper involve the hydrodynamic force (including wall lift and shear lift forces) and the coupling of the hydrodynamic and magnetic torques.

In the absence of a magnetic field, the particle executes symmetric periodic rotations and results in a zero net migration. The particle dynamics were analyzed by varying three parameters: particle aspect ratio, average channel radius, and initial position. We concluded that the elliptical particle rotates and radially oscillates toward and away from the wall but at different rates.

In the presence of a uniform magnetic field, the periodic rotation of the particle becomes asymmetric, and its angular velocity is modified due to the magnetic torque acting on the particle. As a result, the particle radially migrates toward the channel center since the particle rotates backward in different parts of the channel where the magnetic field is stronger. The particle rotates backward in the first half of the curved channel when the magnetic field is applied at the direction $\alpha =0^{\circ }$, whereas a magnetic field applied at $\alpha =90^{\circ }$ results in a backward orientation in the second half of the channel. Furthermore, for an increasing magnetic field strength, the magnetic manipulation on an elliptical particle becomes more prominent for a larger range inside the curved channel, resulting in larger positive radial migrations and velocities.

Different from the analyses of a spherical particle in a low Reynolds number flow in a curved channel by Harding et al. [32,33], a spherical particle, in this case, results in a zero net migration. Therefore, a shape-based separation is feasible by applying a uniform magnetic field. Additionally, we can use previous studies on particle separation in straight channels under uniform magnetic fields, in order to combine both channel geometries for a lab-on-a-chip design. This investigation gives researchers another method for an effective particle shape separation technique for industrial and biological applications.

## Figures and Tables

**Figure A1:**
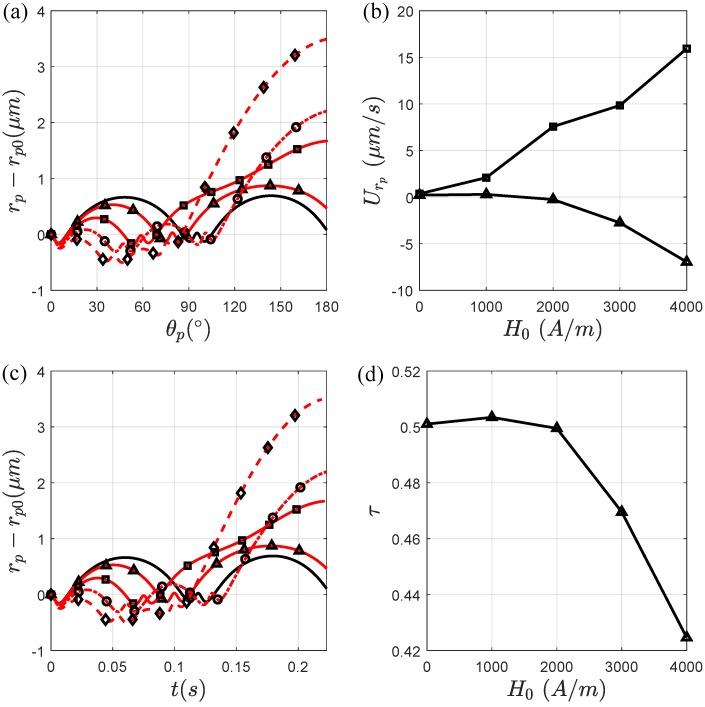
Effect of the magnetic field strength when it is applied at $\alpha =0^{\circ }$ on an elliptical particle (AR=4) that is in the upper curve of the channel. The particle is affected by the magnetic field when its center of mass is approximately at $\theta_{p}=0$. The radial particle-wall distance change of the particle with respect to (**a**) $\theta_{p}$, and (**c**) time for, $H_{0}=0$ A/m (solid black line), $H_{0}=1000$ A/m (triangle symbol), $H_{0}=2000$ A/m (square symbol), $H_{0}=3000$ A/m (circular symbol), $H_{0}=4000$ A/m (diamond symbol). (**b**) The radial velocities $U_{rp}$ (triangle symbol) and $U_{r\theta }$ (square symbol) are functions of $H_{0}$. (**d**) The dimensionless parameter τ as a function of $H_{0}$. We see that as τ decreases, the net migration of the particle decreases.

**Figure A2:**
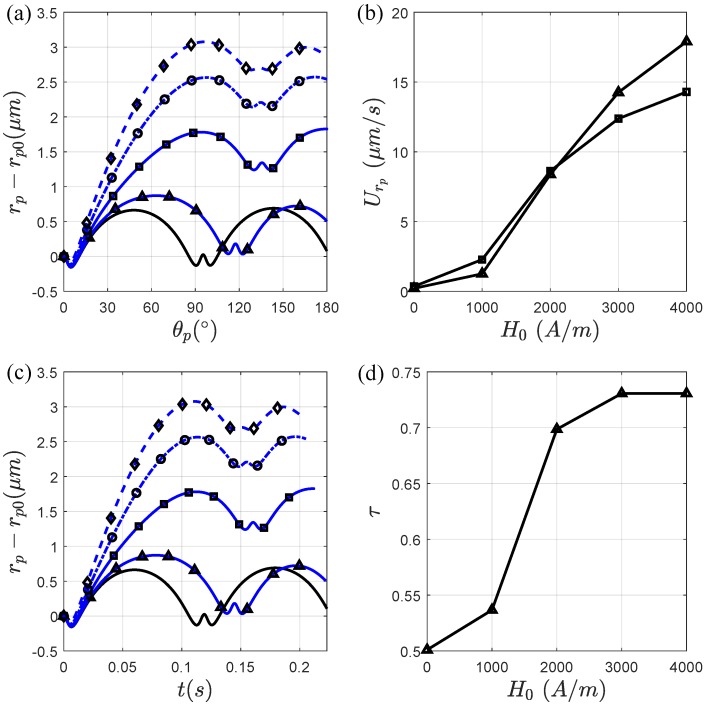
Effect of the magnetic field strength when it is applied at $\alpha =90^{\circ }$ on an elliptical particle (AR=4) that is in the upper curve of the channel. The particle is affected by the magnetic field when its center of mass is approximately at $\theta_{p}=0$. The radial particle-wall distance change of the particle with respect to (**a**) $\theta_{p}$, and (**c**) time for, $H_{0}=0$ A/m (solid black line), $H_{0}=1000$ A/m (triangle symbol), $H_{0}=2000$ A/m (square symbol), $H_{0}=3000$ A/m (circular symbol), $H_{0}=4000$ A/m (diamond symbol). (**b**) The radial velocities $U_{rp}$ (triangle symbol) and $U_{r\theta }$ (square symbol) are functions of $H_{0}$. (**d**) The dimensionless parameter τ as a function of $H_{0}$. We see that as τ increases, the net migration of the particle increases.

**Figure A3:**
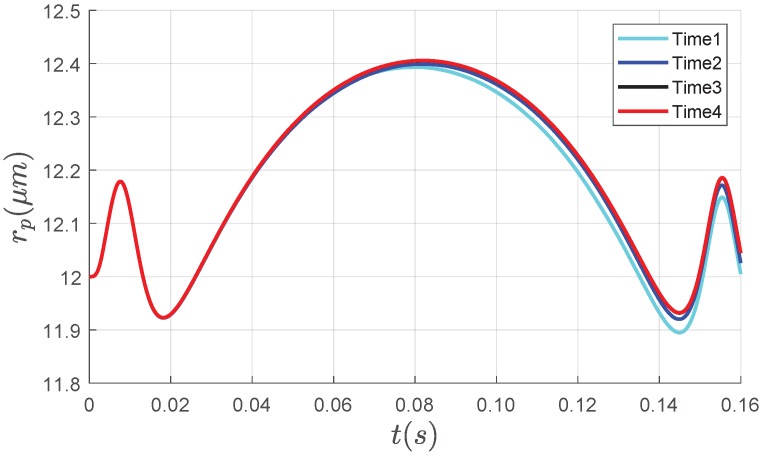
Time independence analysis for a particle’s radial migration as a function of time for AR=4, $r_{p0}=12$ μm, 18,571 Domain Elements, and 152 Particle Surface Elements. The lines for Time 2, Time 3, and Time 4 are very close, which shows that Time 2 still has a reasonable accuracy and a time efficient computation compared to Time 3 and Time 4.

**Figure A4:**
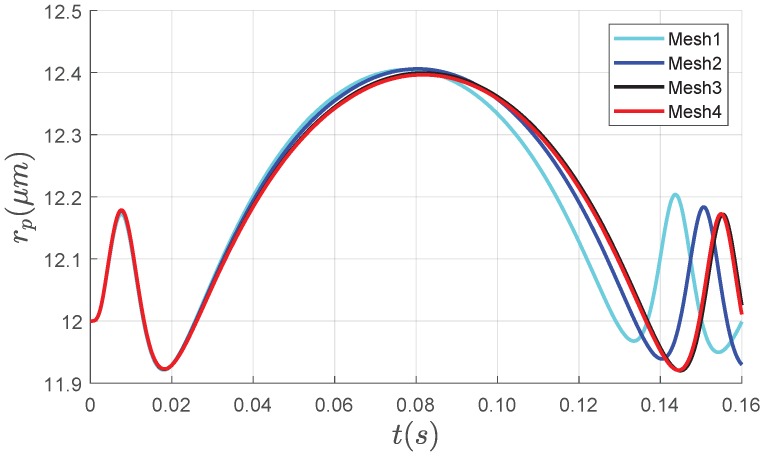
Grid independence analysis for a particle’s radial migration as a function of time for AR=4, $r_{p0}=12$ μm, and a time step $\Delta t=1\times 10^{-5}$ s. The lines for Mesh 3 and Mesh 4 are very close, which demonstrates that Mesh 3 still has a reasonable accuracy and a more efficient computational time.

**Figure A6:**
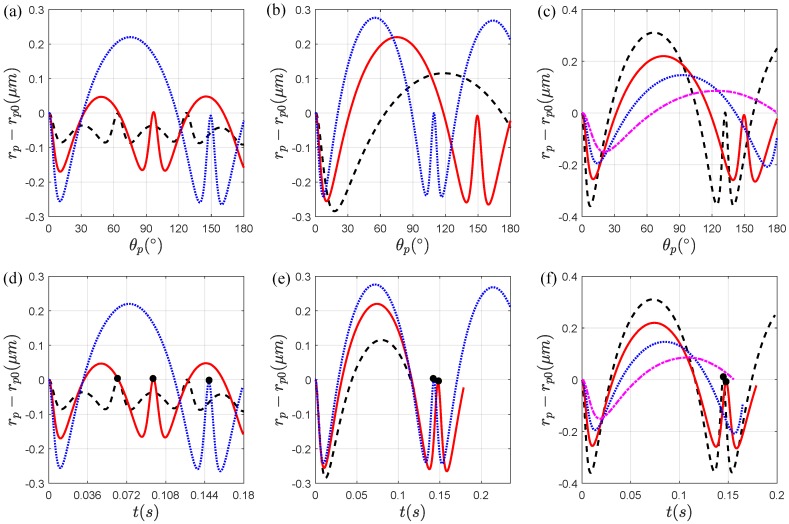
Effects of particle aspect ratio, AR, average radii, $R_{avg}$, and initial radial position, $r_{p0}$, values on the radial particle-wall separation, $\Delta r_{p\pi }$ and $\Delta r_{p\theta }$, of elliptical particles for the range $0^{\circ }\leq \theta_{p}\leq 180^{\circ }$ in the absence of a magnetic field. The (**a**) $\theta_{p}$ and (**d**) time dependency on the aspect ratio effect on the particle’s radial position ($r_{p}-r_{p0}$): AR=2 (dash line), AR=3 (solid line), AR=4 (dot line). The initial position is $r_{p0}=12$ μm and the average channel radius is $R_{avg}=175$ μm. The (**b**) $\theta_{p}$ and (**e**) time dependency on the average radius effect on $r_{p}-r_{p0}$: $R_{avg}=125$ μm (dash line), $R_{avg}=175$ μm (solid line), $R_{avg}=225$ μm (dot line). The particle’s aspect ratio and position are AR=4, $r_{p0}=12$ μm, and respectively. The (**c**) $\theta_{p}$ and (**f**) time dependency of the particle’s initial radial positions on the effect on the radial position $r_{p}-r_{p0}$: $r_{p0}=10$ μm (dash line), $r_{p0}=12$ μm (solid line), $r_{p0}=14$ μm (dot line), $r_{p0}=16$ μm (dash-dot line). The particle’s aspect ratio and the average channel radius are AR=4 and $R_{avg}=175$ μm, respectively. The black dots in (**d**,**e**,**f**) represent the end of the particles’ π rotation.

## Appendix A. Particle in Upper Curve of the Channel

Additional simulations were computed for a particle transporting near the outer curve of the channel ($R_{avg}<∥\left(x_{p},y_{p}\right)∥<R_{out}$). In Figure A1, the effects on the particle dynamics in the absence of a magnetic field and under a uniform magnetic field applied at $\alpha =0^{\circ }$ can be seen, whereas Figure A2 shows a magnetic field applied at $\alpha =90^{\circ }$. In both figures, we investigate the radial migrations, $\Delta r_{p\pi }$ and $\Delta r_{p\theta }$, in terms of (a) $\theta_{p}$, (c) *t*, and the (b) radial velocities, $U_{r\pi }$ and $U_{r\theta }$, and (d) τ in terms of $H_{0}$. We see in Figure A1a,c and Figure A2a,c that, in the absence of a magnetic field, there is no radial migration for $\Delta r_{p\pi }=0$ μm but $\Delta r_{p\theta }>0$ μm, with radial velocities close to zero and τ=0.5. Similar to a particle near the inner curve, even though we see a different velocity profile for a fluid in a curved channel than in a straight channel, as seen in Figure A5e, the particle results in a zero net migration after one periodic rotation. From our data, when τ=0.5, a particle transporting near the lower curve will experience $\Delta r_{p\pi }=0$ μm and $\Delta r_{p\theta }<0$ μm, $\Delta r_{p\pi }=0$ μm and $\Delta r_{p\theta }=0$ μm for a particle transporting in a straight channel, and $\Delta r_{p\pi }=0$ μm and $\Delta r_{p\theta }>0$ μm for a particle transporting near the upper curve.

The effects on a particle radial migration by a magnetic field applied at $\alpha =0^{\circ }$ can be seen in Figure A1. In some cases, the particles complete more than one periodic rotation but their net radial migrations, τ values, and radial velocities are different. As an example, we look at the periodic rotations of the particle for the magnetic field strength $H_{0}=4000$ A/m (diamond symbol) in Figure A1a,c. We see that the first periodic rotation results in a radial migration towards the channel wall but then migrates toward the channel center in the second periodic rotation. The triangle symbol in Figure A1b,d only shows the first periodic rotation but the second periodic rotation can be explained as follows. Since τ needs to be equal to 0.5 for a zero net migration (near the value with the magnetic field strength at 2000 A/m [τ=0.49]), the first periodic rotation for the magnetic field strengths $H_{0}=3000$ A/m and $H_{0}=4000$ A/m are at τ=0.47 and τ=0.42, respectively. The τ values will thus result in $\Delta r_{p\pi }<0$ μm. Therefore, after the second periodic rotation, the τ values are such that $\Delta r_{p\pi }<0$ μm or $\Delta r_{p\pi }>0$ μm. A similar conclusion can be made in Figure A1b where the radial velocities for strengths $H_{0}=3000$ A/m and $H_{0}=4000$ A/m are negative. Thus, $U_{r_{p\pi }}>0$ μm/s after the second periodic rotation for $H_{0}=3000$ A/m, but $U_{r_{p\pi }}<0$ μm/s for $H_{0}=4000$ A/m.

For the overall transportation of the particle in Figure A1b (square symbol) as the magnetic field increases, $U_{r\theta }$ increases and $\Delta r_{p\theta }>0$ μm. An additional observation can be made that the particle near the upper curve radially migrates faster towards the channel center than a particle initially near the lower curve.

The effects on particle net radial migration by a magnetic field applied at $\alpha =90^{\circ }$ can be seen in Figure A2. We notice that in Figure A2a,c the particle radial migration towards the channel center is greater when a particle is near the upper curve than near the lower curve. The radial velocity, for a particle near the outer wall under all magnetic field strengths at the channel curve exit (square symbol), is thus positive and greater than the radial velocity of a particle near the inner wall. Additionally, we see that, for all magnetic field strengths, the particles radially oscillate towards the channel center since τ>0.5 for all magnetic field strengths, as shown in Figure A2d, and the radial velocities are positive and increasing for $U_{r\pi }$ (triangle symbol) and $U_{r\theta }$ (square symbol) in Figure A2b.

## Appendix B. Mesh and Time Step Comparison

The accuracy, convergence, and time efficient calculation of our simulation is based on the time step, number of elements in the computational domain Ω, and the particle surface Γ. To ensure that the time step and the number of elements are efficient for this paper, we compare them by establishing a time and grid independent analysis.

The time independence of an elliptical particle in a curved channel was studied for 18,571 domain elements and 152 elements on the particle boundary as seen in Table A1 and Figure A3. As shown in the plot, the numerical results for the time step $\Delta t=2\times 10^{-5}$ s appears to have a large net migration after one periodic rotation. The time step $\Delta t=1\times 10^{-5}$ s results in a smaller error. The time steps $\Delta t=5\times 10^{-6}$ s and $\Delta t=2.5\times 10^{-6}$ s have very similar results and have a very small error, therefore showing that an elliptical particle results in a zero net migration after one periodic rotation, similar to an elliptical particle in a straight channel. However, the computational time for the latter time steps are longer than for $\Delta t=1\times 10^{-5}$ s. Thus for accuracy and for computational time for all of our simulations, we use the time step $\Delta t=1\times 10^{-5}$ s.

With a time step of $\Delta t=1\times 10^{-5}$ s, we compared the simulation results of four different mesh sizes, in the channel domain and around the particle boundary in a Poiseuille flow and in the absence of a uniform magnetic field are shown in Table A2 and Figure A4. As shown in the plot, the numerical results are efficient enough when the channel domain has 18,571 elements and when there are 152 elements on the particle surface, compared to the lower elements studied. If we increased the number of elements, then there would not be too much difference in the accuracy of the calculations in the simulations. Therefore, in this article, we used 18,571 elements in the computational domain and 152 elements on the the particle surface, thus giving acceptable accurate results for the particle’s transportation and rotation.

**Table A1:** Four time independence analysis for 18,571 computational domain elements and 152 particle surface elements.

Time Step for Time Independence Analysis	Time Step Δt
Time 1	$2\times 10^{-5}$ s
Time 2	$1\times 10^{-5}$ s
Time 3	$5\times 10^{-6}$ s
Time 4	$2.5\times 10^{-6}$ s

**Table A2:** Four meshes for grid independence analysis and for $\Delta t=1\times 10^{-5}$ s.

Mesh for Grid Independence Analysis	Domain Elements	Particle Surface Elements
Mesh 1	11,321	56
Mesh 2	14,020	72
Mesh 3	18,571	152
Mesh 4	24,208	256

## Appendix C. Particle Dynamics Without a Magnetic Field

In this section, we examine the effect of the particle aspect ratio, AR, average radius of the channel, $R_{avg}$, and initial particle-wall separation distance, $r_{p0}$, on the particle dynamics in the absence of a magnetic field. For an elliptical particle in a low Reynolds number and in the presence of a channel wall, a particle will oscillate towards and away from the wall according to a non-zero lift velocity perpendicular to the wall as mentioned by Gavze et al. [35]. These oscillatory motions are caused by some of the forces involved as mentioned by Zhou et al. and the inertia mentioned by Zhang et al. [25,27]. We define the wall lift force as a consequence of the coupling of the rotation and translation dynamics of the particle and the particle-wall hydrodynamic interaction. Consequentially, this force pushes the particle towards the channel center. Conversely, the shear gradient lift force, mentioned in this paper, is due to the non-linearity of the fluid velocity profile and its interaction with the particle. As a result this force pushes the particle towards the wall.

For an elliptical particle in a curved channel and in the absence of a magnetic field, whether or not a particle executes a complete periodic rotation and its net radial migration still needs to be analyzed. The main difference between a particle in a straight channel and in a curved channel is based on the velocity profile and the shear rate due to the channel geometries. The shear rate in a straight channel is applied at the same direction, and its magnitude remains the same so long as the channel width and geometry are constant. As shown in previous studies, an elliptical particle in a straight channel spends an equal amount of time in the first half rotation versus the second half rotation, resulting in a zero net migration [25,26,27,30]. In a curved channel, the shear rate changes directions with respect to the curvature of the channel but the particle net migration still needs to be verified.

Figure A5 shows the dynamics of an elliptical particle with AR=4 initially positioned at $r_{p0}=12$ μm and $\phi_{p0}=43^{\circ }$ and placed in a channel where the average radius is $R_{avg}=175$ μm. As seen in Figure A5a, the particle transportation throughout the curved channel results in a radial migration towards the inner wall, $\Delta r_{p\theta }<0$ μm. Similar to previous research articles, we see that, in Figure A5b, the particle oscillates away from the wall in the first half of its periodic rotation ($0^{\circ }<\phi_{p}^{\prime }<90^{\circ }$) and towards the wall in the second half ($90^{\circ }<\phi_{p}^{\prime }<180^{\circ }$), thus keeping the particle from radially migrating toward the inner channel wall. In Figure A5c, the orientation of the particle, $\phi_{p}^{\prime }$, is a function of the dimensionless time, $t/T_{0}$, where $T_{0}$ is the total time for one periodic rotation. The periodic rotation and the angular velocity, seen in Figure A5d, are symmetric with respect to $\phi_{p}^{\prime }=90^{\circ }$ due to the shear rate. However, even though we have a different channel geometry, the elliptical particle has a net-zero radial migration. In previous works, τ was defined as the ratio between the amount of time the particle spends in the first half of its rotation to its second half [25,26,27,30]. Thus, a particle with AR=4 transporting in a channel with $R_{avg}=175$ μm results in τ=0.50, and the shear gradient lift force acting on the elliptical particle is equal to the second half of its rotation as the wall lift force in the first half, resulting in a zero net radial migration. The radial velocity is also zero, even if we have two different velocity profiles for the curved channel (dashed line) and straight channels (dotted line), as seen in Figure A5e. The wall distance is described as from the inner radius to the outer radius for a curved channel and from the bottom wall to the top wall for a straight channel. We notice that for a straight channel, the velocity profile is symmetric with respect to the channel center (at 25 μm). For the curved channel, however, the velocity profile is asymmetric and the maximum velocity of the flow is between the inner wall and the channel center. However, due to the low inertia acting on the particle, the fluid velocity profile will continue to result in a zero net radial migration.

The net migration of an elliptical particle is shown in Figure A6 for particle aspect ratio, AR, average curve radius, $R_{avg}$, and initial radial particle-wall distance $r_{p0}$. With these different parameters, we can establish an outcome for the radial migration of an elliptical particle after one periodic rotation and upon exiting the curved channel.

In Figure A6a,d, we study how the aspect ratio affects the particle dynamics in a curved channel when $r_{p0}=12$ μm and $R_{avg}=175$ μm. In Figure A6d, as AR increases from 2 to 4, the particle executes fewer periodic rotations in the curved channel because $T_{0}$ increases. Consequentially, for all aspect ratios, $\Delta r_{p\pi }=0$ μm, but they radially migrate towards and away from the inner wall at different rates and the particle completes its π-rotation at different positions inside the curved channel, $\theta_{p}$, even though $r_{p0}$ is the same for all cases. For example, for AR=2, the total time for one periodic rotation is $T_{0}=0.064$ s, $T_{0}=0.096$ s for AR=3, and $T_{0}=0.15$ s for AR=4. Likewise, the periodic rotation is executed earlier in the channel as seen in Figure A6a where $\theta_{p}=64^{\circ }$ for AR=2, $\theta_{p}=97^{\circ }$ for AR=3, and $\theta_{p}=149^{\circ }$ for AR=4. Upon exiting the curve, $\Delta r_{p\theta }=-0.09$ μm for AR=2, $\Delta r_{p\theta }=-0.16$ μm for AR=3, and $\Delta r_{p\theta }=-0.023$ μm for AR=4. The particle radial migration velocity throughout the channel can be observed where a particle with AR=4 will oscillate towards the wall but its overall net migration is less than all the other aspect ratios.

In Figure A6b,e, we increase the average radius $R_{avg}$, while keeping AR=4 and $r_{p0}=12$ μm. For a larger average radius, the particle will complete more periodic rotations since the arc length has increased. The rotational time, $T_{0}$, decreases and the net radial migration after one periodic rotation, $\Delta r_{p\pi }$, is zero for $R_{avg}=175$ μm and $R_{avg}=225$ μm. We see that in Figure A6e, the particle cannot execute a full periodic rotation for $R_{avg}=125$ μm (dash line). In Figure A6b, however, as the particle is exiting the channel curve, $\Delta r_{p\theta }$ increases where $\Delta r_{p\theta }=-0.042$ μm for $R_{avg}=125$ μm, $\Delta r_{p\theta }=-0.023$ μm for $R_{avg}=175$ μm, and $\Delta r_{p\theta }=0.21$ μm for $R_{avg}=225$ μm. As the average radius increases from $R_{avg}=175$ μm to $R_{avg}=225$ μm, $T_{0}$ decreases, as well as the $\theta_{p}$ position at the end of the particle periodic rotation, where $\theta_{p}=149^{\circ }$ for $R_{avg}=175$ μm and $\theta_{p}=109^{\circ }$ for $R_{avg}=225$ μm.

In Figure A6c,f, $r_{p0}$ increases from 10 μm to 16 μm, while keeping AR=4 and $R_{avg}=175$ μm. We see in Figure A6c that as $r_{p0}$ increases, $T_{0}$ also increases since the shear rate is smaller towards the channel center. The radial migration of the particle, however, will experience a zero net radial migration when the particle is placed further away. In Figure A6f, as $r_{p0}$ increases, the particle will less likely perform a periodic rotation and $\theta_{p}$ increases at $\phi_{p}^{\prime }=90^{\circ }$. For particles initially further away from the wall, they spend a shorter time in the channel than all other initial positions shown, but the initial particle-wall distance affects the radial migration of the particle. For all initial positions, the wall lift force is stronger than the shear lift force; thus, $\Delta r_{p\pi }>0$ μm at $\phi_{p}^{\prime }=90^{\circ }$ and increases when $r_{p0}$ decreases. For $r_{p0}=12$ μm and $r_{p0}=10$ μm, the particle can execute a periodic rotation due to the larger shear rate. For $r_{p0}=12$ μm, $T_{0}=0.15$ s and $\theta_{p}=149^{\circ }$ and $T_{0}=0.14$ s and $\theta_{p}=132^{\circ }$ for $r_{p0}=10$ μm for one periodic rotation. At the exit of the channel curve in Figure A6c, the particle will most likely radially migrate towards or away from the inner channel wall but at different rates with $\Delta r_{p\theta }=0.25$ μm for $r_{p0}=10$ μm, $\Delta r_{p\theta }=-0.023$ μm for $r_{p0}=12$ μm, $\Delta r_{p\theta }=-0.095$ μm for $r_{p0}=14$ μm, and $\Delta r_{p\theta }=0$ μm for $r_{p0}=16$ μm. Therefore, in most cases (except at the channel center), the particle experiences a net radial migration towards the inner wall.

The behavior of an elliptical particle migration in a curved channel based on AR, $R_{avg}$, and $r_{p0}$ on $\Delta r_{p\theta }$, and $T_{0}$ can thus be explained as follows. When the particle is in an unbounded fluid domain, the particle freely rotates because there is no inertia acting on the particle [34]. Thus, there is no net lateral migration. When the channel wall is present, there is a particle-wall hydrodynamic interaction that increases the resistance on the particle’s rotation [35]. As the particle aspect ratio increases, the periodic rotation of the particle increases because the particle-wall hydrodynamic interaction is more significant for larger aspect ratios than for smaller aspect ratios. Therefore, even if the shear lift force is greater than the wall lift force, a particle with a smaller aspect ratio radially migrates towards and away from the wall faster than particles with a larger aspect ratio. Next, as the average radius increases, the particle completes a periodic rotation faster and at a shorter $\theta_{p}$. For the first half of its rotation, $\Delta r_{p\pi }$ from $\phi_{p}^{\prime }=0^{\circ }$ to $\phi_{p}^{\prime }=90^{\circ }$ increases, indicating that the channel geometry affects the particle dynamics during its transportation throughout the channel curve. Finally, as $r_{p0}$ decreases, the wall lift force on the particle becomes more prominent since the shear rate is also larger near the channel wall. When a particle is initially placed further away from the inner wall, the shear lift force has a larger impact on the radial migration except at $r_{p0}=25$ μm. Even though the last two parameters have been studied for AR=4, we can make a similar conclusion for AR=2 and AR=3.

We can, therefore, make a few conclusions and predictions for an elliptical particle transporting in a curved channel in the absence of a magnetic field. First, the channel geometry, the particle-wall separation distance, and the particle aspect ratio are important factors that ultimately affect the rotation, the radial oscillatory motion, and the overall channel transportation of the particle in a curved channel. Second, for a radial oscillatory motion after one periodic rotation, $\Delta r_{p\pi }=0$ μm at $\phi_{p}^{\prime }=180^{\circ }$, but the particle-wall distance is positive, $\Delta r_{p\pi }>0$ μm, at $\phi_{p}^{\prime }=90^{\circ }$. The transportation of the particle throughout the curved channel is affected since the particle continues to radially oscillate, but the particle migrates either toward or away from the inner wall. In these cases, the shear lift force may be equal to the wall lift force and the radial migration depends on the rotational dynamics of the particle.
